# Links between metabolic plasticity and functional redundancy in freshwater bacterioplankton communities

**DOI:** 10.3389/fmicb.2013.00112

**Published:** 2013-05-09

**Authors:** Jérôme Comte, Lisa Fauteux, Paul A. del Giorgio

**Affiliations:** ^1^Département des Sciences Biologiques, Groupe de Recherche Interuniversitaire en Limnologie, Université du Québec à MontréalMontréal, QC, Canada; ^2^Département de Biologie, Centre d’Études Nordiques, Unité Mixte Internationale Takuvik, Institut de Biologie Intégrative et des Systèmes, Université LavalQC, Canada

**Keywords:** freshwater bacterioplankton, metabolic plasticity, traits selection, functional redundancy, community composition, environmental forcing

## Abstract

Metabolic plasticity and functional redundancy are fundamental properties of microbial communities, which shape their response to environmental forcing, and also mediate the relationship between community composition and function. Yet, the actual quantification of these emergent community properties has been elusive, and we thus do not know how they vary across bacterial communities, and their relationship to environmental gradients and to each other. Here we present an experimental framework that allows us to simultaneously quantify metabolic plasticity and functional redundancy in freshwater bacterioplankton communities, and to explore connections that may exists between them. We define metabolic plasticity as the rate of change in single-cell properties (cell wall integrity, cell size, single-cell activity) relative to changes in community composition. Likewise, we define functional redundancy as the rate of change in carbon substrate uptake capacities relative to changes in community composition. We assessed these two key community attributes in transplant experiments where bacterioplankton from various aquatic habitats within the same watershed were transplanted from their original water to waters from other systems that differ in their main resources. Our results show that metabolic plasticity is an intrinsic property of bacterial communities, whereas the expression of functional redundancy appears to be more dependent on environmental factors. Furthermore, there was an overall strong positive relationship between the level of functional redundancy and of metabolic plasticity, suggesting no trade-offs between these community attributes but rather a possible co-selection. The apparent continuum in the expression of both functional redundancy and plasticity among bacterial communities and the link between them, in turn suggest that the link between community diversity and function may also vary along a continuum, from being very tight, to being weak, or absent.

## INTRODUCTION

There is now ample evidence that the overall metabolic performance of aquatic bacterial communities is mainly driven by environmental factors in a manner that is roughly predictable (Ducklow, 2008; Lennon and Cottingham, 2008; Comte and del Giorgio, 2009), and yet there is much unexplained variation in the response of microbes to environmental changes (e.g., Comte and del Giorgio, 2009). The extent to which the composition and diversity of bacterial communities play a role in shaping their overall performance and their responses to environmental forcing has been a focus of research in aquatic microbial ecology (Langenheder et al., 2005, 2006, 2010; Findlay and Sinsabaugh, 2006; Bertilsson et al., 2007; Gamfeld and Hillebrand, 2008; Boucher and Debroas, 2009; Lindström et al., 2010; Comte and del Giorgio, 2010, 2011; Peter et al., 2011; Reed and Martiny, 2013). Results from both field studies (Shade et al., 2007; Jones et al., 2009; Nelson, 2009) and laboratory experiments (Judd et al., 2006; Kritzberg et al., 2006; Newton and McMahon, 2011) have shown, for example, that shifts in the nature or the source of organic matter can induce changes both in community composition and in various aspects of community metabolism, yet it is still uncertain to what extent the actual metabolic response may be mediated by these changes in community structure, or whether the two simply covary.

One of the reasons it has been so difficult to establish clear links between bacterial community composition (BCC) and the functional and metabolic responses of the community to environmental forcing is that this link is not direct, but rather may be mediated by aggregate properties of these communities. One such aggregate property is the degree of metabolic plasticity that exists at the community level (also termed community “resistance” by Allison and Martiny, 2008), which reflects the capacity of a community to accommodate environmental changes by adjusting the overall performance of existing dominant phylotypes.

Another key emergent community property that may mediate community composition and overall response is functional redundancy, which implies that different phylotypes can perform similar functional role in the community. Therefore, this emergent community property may explain the reported lack of connection between community composition and key aspects of bacterial community performance and function, as well as the fact that stable ecosystem function is generally maintained under very different configurations of community composition (e.g., Fernàndez et al., 1999; Langenheder et al., 2005). Although there is debate on whether functional redundancy is high (Wohl et al., 2004) or moderate (Peter et al., 2011) in microbial communities, the reality is that this community property has seldom been quantified, and we do not know how functional redundancy varies among microbial communities.

These community-level properties are key for two reasons: They influence the response of bacterial communities to environmental forcing, and on the other hand, they modulate the relationship that may exist between community composition and community function and performance. Lack of correlation between features of bacterial function and of composition has traditionally been interpreted as evidence of a high degree of functional redundancy (e.g., Rosenfeld, 2002; Allison and Martiny, 2008). In contrast, significant relationships between these variables are usually interpreted as diversity and composition having a significant influence on community performance (and therefore on ecosystem functioning), suggesting in turn that functional redundancy may be more constrained.

Likewise, there is ample evidence that individual aquatic bacterial taxa may be extremely flexible in terms of the breadth of physiological and morphological adjustments to their environment (Hahn et al., 2003; Jaspers and Overmann, 2004; Meyer et al., 2004; Buchan et al., 2005; Hahn, 2006; Walker et al., 2006; Schimel et al., 2007; Allison and Martiny, 2008; Comte and del Giorgio, 2011). However, metabolic plasticity at the community level, aka community resistance (Allison and Martiny, 2008), like functional redundancy, is a difficult concept to quantify, and we still do not know its magnitude and variability among communities.

These emergent properties, i.e., plasticity and redundancy, result from the sum of the physiologic and life history traits of the ensemble of individual players within the community, but the scaling of these individual traits to the community level is still not well understood (Bohannan et al., 2002; Suding et al., 2008; Hillebrand and Matthiessen, 2009; Shade et al., 2012). As with species or ecotype traits, we can question whether there are trade-offs associated to these two community-level traits, or whether they are in fact co-selected by the environmental and the biological forcing factors that shape the structure of these communities. The rules of co-selection and trade-offs associated to traits within a single-organism, however, may not apply to community-level features that emerge from the combination of individual traits (e.g., Suding et al., 2008; Hillebrand and Matthiessen, 2009). For example, we can hypothesize that communities that are intrinsically more plastic may be less functionally redundant, because there may be trade-offs at the individual level between the breadth of physiologic tolerance and flexibility, and the breadth of resource utilization. Moreover these trades-off can vary along environmental gradients as previously shown for other microbial traits (Jessup and Bohannan, 2008). These questions have not been addressed for natural aquatic bacterial communities.

There are thus major conceptual and technical challenges associated to these two community properties, and although they have been extensively discussed and invoked in contemporary microbial ecology, they have seldom, if ever, been actually quantified and compared. We do not know how they vary among communities, how they are regulated, if they are ecosystem-specific, and more importantly, we do not know what the relationships and trade-offs are between these two key properties of microbial communities. In this paper we explicitly address these issues in freshwater bacterioplankton communities.

### CONCEPTUAL FRAMEWORK

Bacterial plasticity is linked to the average breadth of morphological and physiological characteristics of cells affecting their individual performances such as growth, cell division, and respiration. For example, there is clear evidence that bacteria can respond to shifts in environmental conditions by modifying their size, physiology and activity, which may ultimately lead to dormancy (Schimel et al., 2007; Lennon and Jones, 2011; Evans and Hofmann, 2012) or tolerance to environmental perturbations (Meyer et al., 2004). In this context, high community plasticity is associated to shifts in single-cell properties with little or no change in community composition, and therefore to communities that can accommodate environmental change with physiological and morphological adjustments of the dominant phylotypes. In our framework, community plasticity can be quantified as changes in these single-cell characteristics (SCC), evidenced as shifts in a host of individual properties of cells measured by flow cytometry (cell wall integrity, cell size, single-cell DNA content, and activity), relative to changes in BCC. In practice, this can be quantified as the slope of the regression model of SCC as a function of BCC under circumstances where both SCC and BCC are varying, for example, in time within a given site, in space along natural environmental gradients, or under experimental manipulations of environmental factors. The change in SCC and in BCC under these circumstances can be quantified in terms of dissimilarity between successive states of the same community, and high values of slopes between the dissimilarities observed in SCC relative to those in BCC would indicate a high degree of plasticity for that particular community, and vice versa.

Redundancy, on the other hand, is linked to the level of overlap in functional capacities (FCs) among the dominant phylotypes, such that different taxa provide similar functions to the community (Burke et al., 2011). In our experimental framework, functional redundancy is defined as the magnitude of change in FCs relative to the magnitude of change in community composition, again under circumstances where both FC and BCC are varying (as for SCC above); we use the organic substrate uptake profiles of the community as a measure of FC. In this framework, small changes, and therefore a high level of similarity in FC together with stronger changes in BCC, for example along an environmental gradient, would indicate a high level functional redundancy within the community.

**FIGURE 1:**
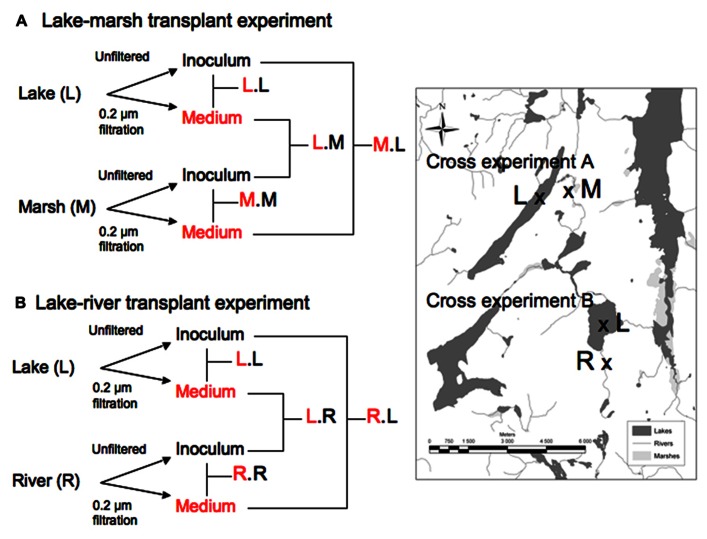
**Experimental design of the study – Two-way factorial design with two series of experiments: Lake Bowker-Marsh (A) and Lake Fraser-River (B) Each experiment was replicated two times (June and July 2006).** Samples were taken at days 0, 2, 3 and 5. L, M, R correspond to lake, marsh and river respectively. The treatment assignment presents the source of medium first (in red) and the source of the inoculum.

We have applied the above metrics to assess the extent of metabolic plasticity and functional redundancy among local communities within same regional metacommunity, and how these two emerging properties relate to each other, in experiments where we manipulated the environment in order to allow these communities to express their intrinsic levels of metabolic plasticity and functional redundancy. Rather than exposing these bacterial communities to artificial conditions, we chose to use reciprocal transplant experiments, which have been commonly used to test for effects of both environment and composition (and their interactions) on the functioning of microbial communities in multiple environments (e.g., Gasol et al., 2002; Kirchman et al., 2004; Langenheder et al., 2005; Reed and Martiny, 2007, 2013; Strickland et al., 2009; Bell, 2010; Shade et al., 2010), and wherein local freshwater bacterial communities were grown in their own original water, and also in water originating from other habitat types that exist within the same watershed (**Figure 1**). We transplanted lake bacterial communities into river water and vice versa, and lake bacterial communities into marsh waters and vice versa, and these environments differed greatly in terms of chemistry and organic matter (**Table 1**). These transplant experiments allowed us to derive two alternative but complementary estimates of both plasticity and redundancy. On the one hand, we calculated an “absolute” measure of functional redundancy and plasticity in both controls and transplanted communities, and used these to test whether redundancy and plasticity are intrinsic features of these communities. On the other, we estimated a more integrative measure of both bacterial communities properties (i.e., “relative plasticity and redundancy”), where plasticity and redundancy measured in transplanted communities were calculated relative to control communities. We derived these metrics as follows:

**Table 1:** Characteristics of the aquatic ecosystems sampled

**Site**	**DOC (mg L^–1^)**	**TP (μg L^–1^)**	**TN (mg L^–1^)**	**Temp (°C)**	**Cond (mS cm^–1^)**	**BP (μg C L^–1^h^–1^)**	**BA (10^6^ mL^–1^)**
Lake Bowker	2.1 ± 0.05	2 ± 1.04	0.14 ± 0.02	22.1 ± 0.8	0.056 ± 0.01	0.21 ± 0.07	1.5 ± 0.1
Marsh	5.1 ± 0.46	7.1 ± 1.5	0.26 ± 0.02	21.8 ± 1.3	0.057 ± 0.01	1.15 ± 0.05	4.1 ± 1.9
River	10.2 ± 1.7	21.6 ± 1.2	0.45 ± 0.02	16.8 ± 3	0.046 ± 0.02	2 ± 0.63	3.7 ± 1.3
Lake Fraser	6 ± 0.38	6 ± 1.9	0.22 ± 0.03	19.3 ± 1.8	0.057 ± 0.01	1.41 ± 0.4	4.2 ± 1.5

*Values represent in situ mean (±standard deviation) concentrations of dissolved organic carbon (DOC), total phosphorus (TP) and total nitrogen (TN), average water temperature (Temp) and conductivity (Cond), mean rates of bacterial production in terms of ^3^H-leucine uptake (BP) and bacterial abundance (BA).*

**FIGURE 2:**
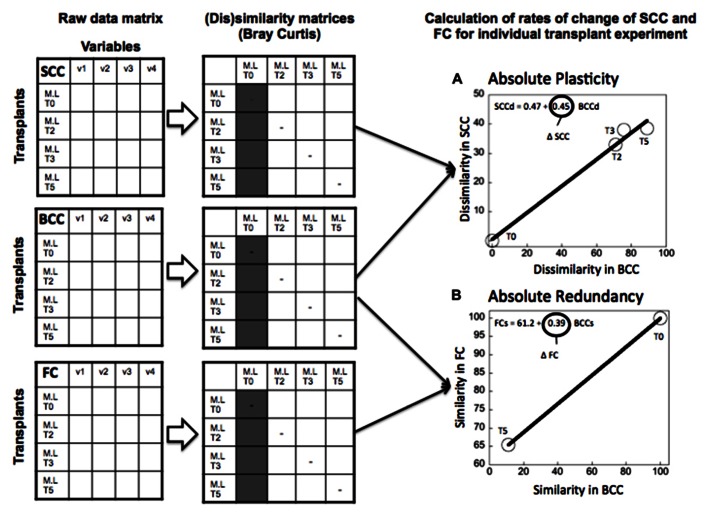
**Example of calculation of the “absolute” plasticity (A) and“absolute” functional redundancy (B) for the transplant experiment (June 2006), where lake bacteria were inoculated in marsh water.** Functional redundancy (Δ FC) was calculated as the slope of the relationship between the rate of change in similarity (Bray Curtis) of functional capacities at the different time points of the experiment relative to the community at time 0 of the experiment (marked in gray in the similarity matrix), and the corresponding changes in similarity in community composition (BCCs). Metabolic plasticity (Δ SCC) was estimated as the slope of the relationship between the dissimilarity in single-cell characteristics (SCCd) relative to the community at time 0, and the corresponding changes in the relative dissimilarity in BCC (BCCd) over the same period. Each circle represents the average similarity in FC (or dissimilarity in SCC) per unit similarity (or dissimilarity) in BCC at each time point of the experiment. Dissimilarity and similarity indices were estimated using Bray Curtis metric. The line represents the least square regression fit and the slope of the regression model refers to either Δ FC or Δ SCC.

**FIGURE 3:**
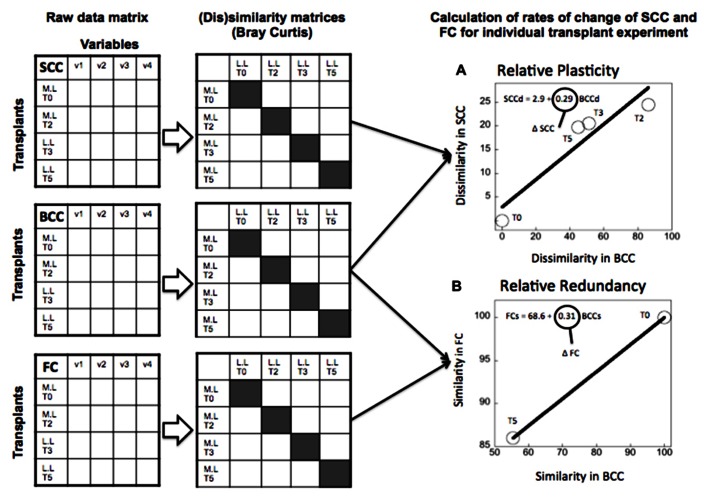
**Example of calculation of the extent of “relative” plasticity (A) and “relative” functional redundancy (B) for the transplant experiment (June 2006) where lake bacteria were inoculated in marsh water.** Functional redundancy (Δ FC) was calculated as the rate of change in community functional capacities similarity (FCs) relative to similarity in community composition (BCCs) between control and transplanted communities at each time point of the experiment (highlighted in gray in the similarity matrix). Metabolic plasticity (Δ SCC) was estimated from the rates of changes in dissimilarity in single-cell characteristics (SCCd) relative to BCC dissimilarity (BCCd) over time. Each circle represents the average similarity in FC (or dissimilarity in SCC) per level of similarity (or dissimilarity) in BCC at each time point of the experiment. Dissimilarity and similarity indices were estimated using Bray Curtis metric. The line represents the least square regression fit and the slope of the regression model refers to either Δ FC or Δ SCC.

(1) “Absolute” plasticity and redundancy (**Figure 2**): For each individual treatment we estimated the rate of change in SCC, FC, and BCC over time, and then combined these to derive plasticity (as the slope of SCC vs BCC) and redundancy (as the slope of the FC vs BCC). We did this for the community incubated in its original water, and the same community incubated in a different source of water, such that we had two separate estimates of plasticity and redundancy for any given community. This allowed us to test whether there was a correlation between plasticity and redundancy expressed under different environmental scenarios, and therefore, whether these properties are intrinsic to the community or whether they are driven by environmental forcing.
(2) “Relative” plasticity and redundancy (**Figure 3**): In the second approach, we followed the change in SCC, FC, and BCC not between time points within a given treatment, but rather between the control and the transplanted samples of the same community. This approach yielded a single estimate of plasticity (also as change in SCC relative to change in BCC), and redundancy (as change in FC relative to change in BCC) per community, which is based on the breadth of response in SCC, FC, and BCC when the community is taken outside its native environment. This alternative estimate provides a more integrative quantification of plasticity and redundancy that takes into account the background variability that these communities express in their native environment, and which therefore highlights their response to environmental forcing.

## MATERIALS AND METHODS

### SAMPLING AND EXPERIMENTAL SET-UP

The experiments involved two lakes, a headwater river and a freshwater marsh, all located within the same temperate watershed in southeastern Québec (45.508N, 73.588W). These systems are part of the same hydrological network but differ in their limnological characteristics (**Table 1**).

The experimental design was the following: we carried out two series of transplants: The first series (Experiment A), consisted of (i) inoculating the upstream oligotrophic lake water (lake Bowker) with bacteria originating from the downstream marsh (L.M indicating that lake water received marsh bacteria), (ii) inoculating marsh water with lake bacteria (i.e., M.L), (iii) inoculating each medium with its own microbial assemblages (L.L and M.M) (**Figure 1**, top panel). The second series (Experiment B), consisted of (i) inoculating lake water (Lake Fraser) with the inflowing river bacteria (L.R); (ii) inoculating the upstream river water with bacteria from the receiving lake Fraser (R.L); (iii) inoculating each medium with its own microbial assemblages (L.L and R.R; **Figure 1**, bottom panel). Controls with only medium but no inoculum were also prepared for each experiment. We carried out these two experiment series twice, in June and July of 2006, so that there are a total 8 transplant experiments with their respective controls.

The transplant experiments were carried out using dialysis bags (Spectrum labs, MWCO: 12–14kDa). The bags were cut to accommodate a volume of 600ml, and were thoroughly washed, rinsed and soaked in Nanopure water before use. Water for media was prepared by sequentially filtering the sample through 3μm precombusted glasfiber filters (A/D filter, Pall Corporation), and 0.2μm filter Capsule (Acropak 1000 supor capsule membrane, Pall Corporation) to remove most of the ambient organisms; this medium was then inoculated with unfiltered water (1% vol/vol), and the bags were sealed with clamps. We prepared a total of six bags for each treatment, which were incubated submerged in a tank filled with 40L of the corresponding unfiltered medium water, kept in the dark at 20°C. The tank water was renewed at day 3 to maintain this water as close to ambient as possible. We took samples at time zero, and then removed duplicate bags at time = 2, 3, and 5days for further analyses.

### BIOLOGICAL VARIABLES

Bacterial communities metabolism was assessed as rates of incorporation of ^3^H-leucine following protocol described in Kirchman (1993). We used the profile of carbon substrate utilization, measured from BIOLOG Ecoplates as a proxy for community FC. For logistical purpose, measures were taken at the beginning and end of each incubation only. The plates were inoculated with the water samples and incubated in the dark at room temperature, and the absorbance was recorded in a microplate reader (Tecan GENios) every day for 5days. We used the time at which the average well color development (AWCD) was closest to the reference absorbance of 0.5, as the end point (Garland et al., 2001). We measured several SCC using flow cytometry (FACScalibur, Becton Dickinson) at each sampling time points (time = 0, 2, 3 and 5days). Total bacterial abundance, and the abundance of high and low DNA populations (HNA and LNA respectively) was determined using SYTO 13. Respiring and dead cells were enumerated using CTC and Live/Dead kit respectively, in addition to cells with compromised (DiBAC4) and intact (DiOC6 (3)) membranes. In all cases, we used the average fluorescence values for each of the assays (from the different fluorochromes) as well as the average side scatter as measures of single-cell properties. BCC was determined by denaturing gradient gel electrophoresis (DGGE). DNA was extracted using CTAB buffer and chloroform/isoamyl alcohol. PCR reactions were performed using GC clamp-358 F and 907rM primers (HPLC purified, Sigma Genosys). DGGE gels were build on 100 ng of DNA and ran for 16 h at 100V and 60°C on 40–65% acrylamide gels and analyzed using Quantity one software (Biorad). All procedures described above are detailed in Comte and del Giorgio (2009).

### CONSTRUCTION OF RAW DATA AND DISSIMILARITY MATRICES

We constructed raw data matrices for each of the three components considered in the study (FC, SCC, and BCC), where rows represent the different treatments at the different time points during the experiment, and columns correspond to averages (from the duplicate bags) of the variables measured for each component (SCC, FC, and BCC) (see conceptual **Figures 2** and **3**). In the case of the BCC matrix, each column corresponds to the relative contribution of each band to the overall fluorescence of the sample. The matrix of SCC consisted of the average fluorescence and side-scatter estimates obtained from the cytometric analyses. In the case of the FC matrix, each column represents the absorbance values for each of the 31 substrates that are included in the Ecoplates. For each raw matrix, data were log_10_-transformed, (except for the BCC data, which were arcsine transformed), normalized, and standardized. We then generated a dissimilarity matrix (for the estimation of plasticity based on SCC and BCC), or a similarity matrix (for the estimation of redundancy based on FC and BCC) in both cases based on the Bray Curtis metric (Primer 5.2 software). We chose the Bray Curtis metric because it is bound between 0 and 100, which allowed us to compare the above three components: For example, a Bray Curtis dissimilarity value of 0 means that two communities have the same composition (or FC or SCC), and 100 means the two communities do not share any phylotypes or any SCC or FC features.

### CALCULATION OF BACTERIAL PLASTICITY AND FUNCTIONAL REDUNDANCY

As described previously, we produced two alternative estimates of plasticity: (1) We regressed the dissimilarity in SCC and in BCC in time for each individual treatment, and used the resulting slope as a measure of “absolute” plasticity (**Figure 2A**). (2) We calculated the dissimilarity in SCC between the transplanted and the control community at each time point, and we regressed this against its counterpart in BCC, and used the slope as a measure of “relative” plasticity (**Figure 3A**). We proceeded likewise to estimate “absolute” and “relative” redundancy (**Figures 2B** and **3B**), except that we used similarity rather than dissimilarity so as to obtain a more intuitive positive relationship between FC and BCC, such that higher slopes imply higher redundancy and vice versa. The relationship between plasticity and functional redundancy was investigated using least square regression model (Jmp 7.0).

## RESULTS

### HETEROGENEITY IN ECOSYSTEMS CHARACTERISTICS

The aquatic ecosystems sampled differed greatly in terms of their limnological characteristics (e.g., conductivity, nutrients, and dissolved organic carbon concentration). These differences in physicochemical variables coincide with differences in the total abundance and biomass production rates of bacteria as assessed by the uptake of ^3^H-leucine (**Table 1**).

### VARIABILITY IN PLASTICITY AND REDUNDANCY BETWEEN AND WITHIN COMMUNITIES

**FIGURE 4:**
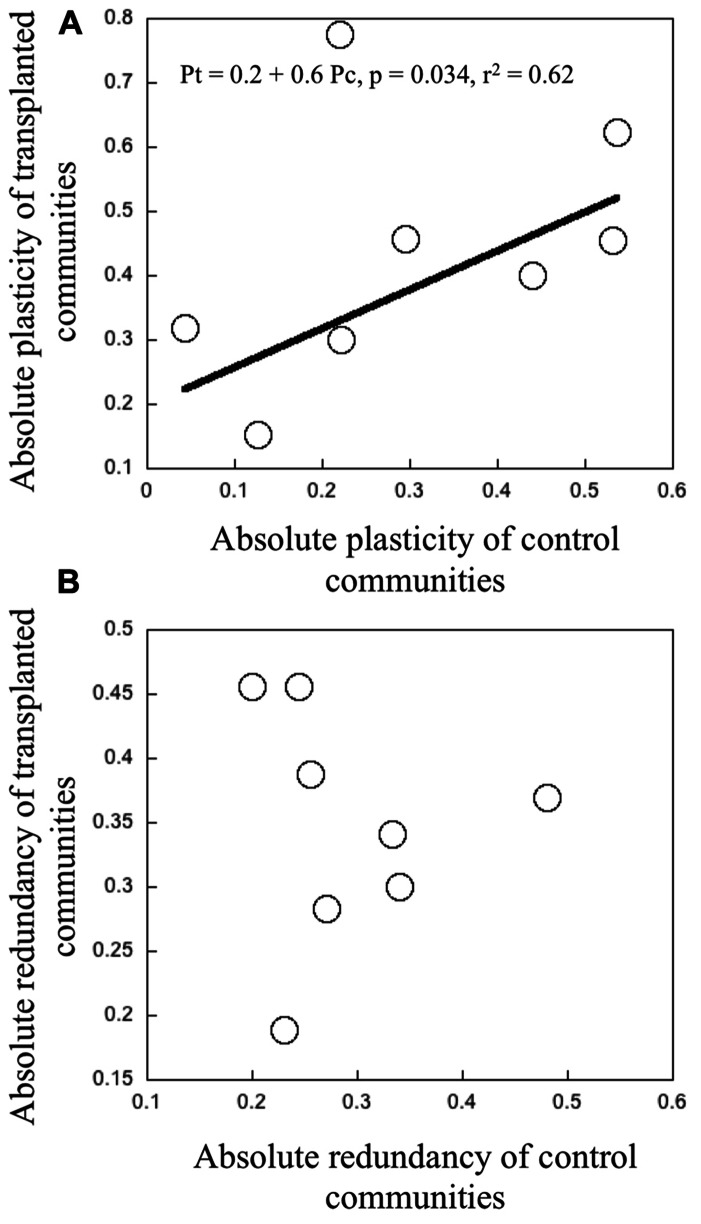
**(A)** Relationship between the “absolute” plasticity in transplanted communities as a function of “absolute” plasticity in control communities. **(B)** The relationship between the “absolute” redundancy measures in transplanted and control communities. Each point represents the average magnitude of plasticity and redundancy per treatment of transplant experiments in June and July 2006. The black line represents the fit from a least regression model with a significant threshold at p < 0.05.

We found a relatively large range in “absolute” plasticity, both among different communities, and also within a given community, between the control and the transplanted treatments, the latter generally having higher values. There was a significant positive relationship between the “absolute” plasticity in the controls and in the transplanted treatments for any given community (*r*^2^ = 0.62, *p* = 0.034, **Figure 4A**). Bacterial communities that had high “absolute” plasticity when grown in their native environment, also tended to express high plasticity when transplanted into a different environment, and vice versa. There was one clear outlier that corresponds to experiment B conducted in June, in which bacterial assemblage from lake Fraser were inoculated in the water from the inflowing river. However, there is no explanation for it. “Absolute” redundancy also showed a wide range, both among and within communities, but as opposed to plasticity, there was no relationship at all between the “absolute” redundancy in the controls and in the transplanted communities (**Figure 4B**).

### RELATIONSHIP BETWEEN COMMUNITY PLASTICITY AND FUNCTIONAL REDUNDANCY

**FIGURE 5:**
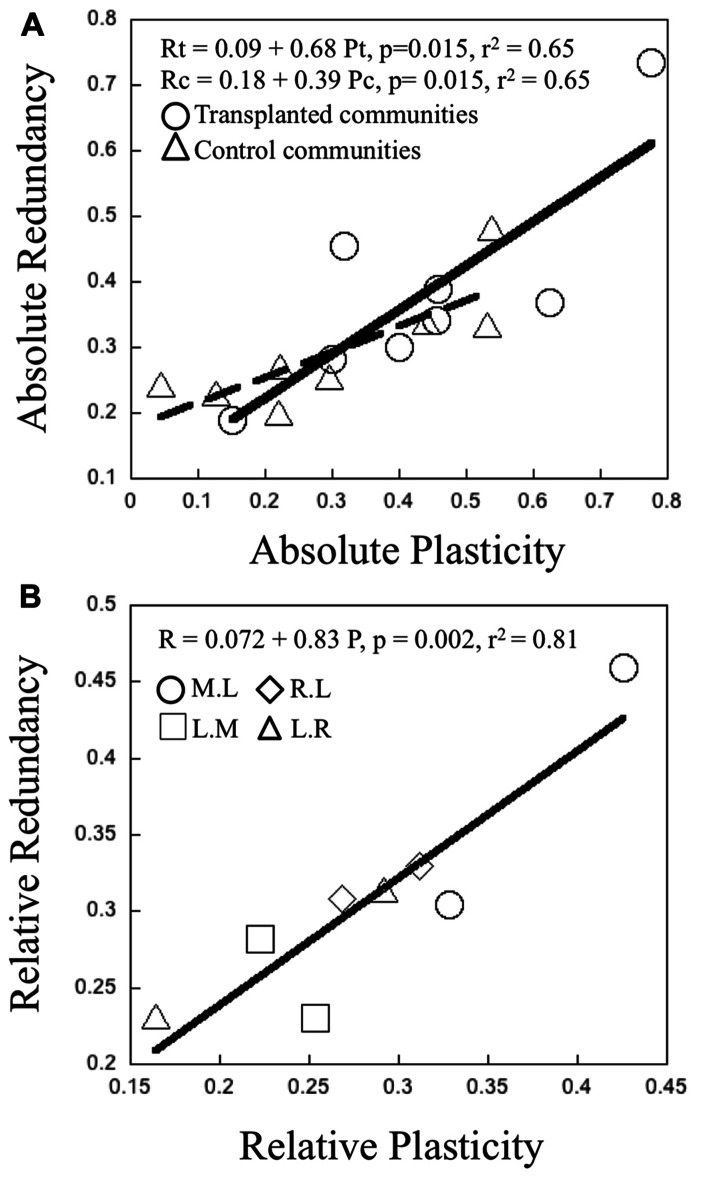
**(A)** Relationship between the magnitude of “absolute” community functional redundancy and “absolute” bacterial metabolic plasticity. Each point represents the average plasticity and redundancy per treatment of transplant experiments in June and July 2006. Open circles and triangles refer to transplanted and control communities respectively. The black full and dashed lines represent the trend generated from a least regression model (*p* <0.050 for transplanted and control communities respectively. **(B)** Relationship between “relative” community functional redundancy and “relative” bacterial metabolic plasticity. Each point represents the average magnitude of plasticity and redundancy per treatment of transplant experiments in June and July 2006. Open circles, squares, diamonds, and triangles refer to M.L, L.M, R.L, and L.R treatments respectively. The black line represents the trend generated from a least regression model (p < 0.05).

There was a significant positive relationship between the “absolute” plasticity and “absolute” redundancy, both for the control and transplant communities (**Figure 5A**). The latter had overall higher values for both variables, as was to be expected. There was an even stronger positive relationship between “relative” plasticity and “relative” redundancy (**Figure 5B**). Overall, communities characterized by high intrinsic metabolic plasticity (absolute and relative), also tended to express higher functional redundancy and vice versa. There was a tendency for lake communities to have higher overall “absolute” and also “relative” plasticity and redundancy, and for rivers to have the lowest, but these differences were not statistically significant.

## DISCUSSION

The relationship between biodiversity and ecosystem functioning has generated much interest and debate in recent years. There are two main lines of thought in this regard, the first increasing the number of species (increasing richness) should result in increased ecosystem functioning and/or stability (e.g., Bell et al., 2005; Horner-Devine et al., 2006), and the second is that patterns in microbial function should correlate with the presence or absence of specific species or phylotypes (e.g., Peter et al., 2011). In both conceptual frameworks, functional redundancy and metabolic plasticity are relegated to serving as null hypotheses (Naeem, 1998; Loreau, 2004). These two community traits play a key role in influencing the response of bacterial communities to environmental forcing and in modulating the relationship between community composition and function. Although they have often been evoked and discussed, they remain however difficult to quantify in natural communities, and their actual patterns within and across bacterial communities, and more importantly their relationship to each other have seldom been explored.

In previous studies (Comte and del Giorgio, 2011), we had hypothesized that metabolic plasticity plays no role in the actual outcome in terms of metabolism, but does play a role in terms of the pathways of response to environmental forcing, because this property determines whether the community response is mediated by changes in composition or not. We also hypothesized that both metabolic plasticity and functional redundancy are intrinsic, emerging properties of bacterial communities, which do not depend on the environmental conditions but rather is determined by community composition. In this paper we explicitly addressed these hypotheses, and in addition, we have explored the potential connections that might exist between these two fundamental community properties.

In order to address these questions, we first had to develop a conceptual framework and an experimental approach, which would allow us to quantify these properties and empirically test their patterns. There are no doubt drawbacks in our approach. For example, substrate uptake profiles are only one aspect of bacterial function, and these results thus cannot be directly extrapolated to other aspects of function. Likewise, we used single-cell properties to derive a metric of metabolic plasticity, but there are many other dimensions to bacterial metabolism that are not considered here. Nevertheless, we feel that the approach does provide quantitative metrics for both metabolic plasticity and functional redundancy that can be compared among samples and between each other.

The first major question we addressed is how redundancy and plasticity vary within and among bacterial communities. In particular, we explored whether these properties are driven by environmental factors, or whether they can be considered intrinsic features of freshwater bacterial communities, in the sense that a given community will consistently show a high or a low degree of redundancy and plasticity under different environmental scenarios. Our results using our “absolute” metrics suggest that plasticity is indeed an intrinsic feature of these communities, because there was a strong relationship between the level of plasticity expressed by any given community under two very different environmental scenarios, with communities that express a high level of plasticity in one environmental scenario tend to also express high plasticity when exposed to a different environment, and vice versa. If the expression of community plasticity were entirely environmentally-driven, we would not necessarily expect such positive relationship, and that was in fact the case for functional redundancy. We also found significant variability in functional redundancy, but the lack of relation between control and transplant treatments would suggest that the expression of this property might be more strongly controlled by environmental factors, such as the nature of the DOC pool or other environmental drivers. If this is the case, it would suggest that all bacterial communities may have a high potential for functional redundancy, but that the expression of this property is directly related to environmental forcing or other factors.

Although there was a consistent pattern of plasticity within a given community, it is worth pointing out that the expression of plasticity for a given community somewhat differed between the two environmental scenarios, the magnitude being lower in the controls relative to the transplants as would be expected, suggesting that although this appears to be an intrinsic community trait, there is nevertheless an environmental component in the expression of this trait. The expression of functional redundancy, on the other hand, appears to be more strongly driven by environmental factors, and there was no consistent pattern in the expression of redundancy under the two environmental scenarios. These findings in turn suggest that the distribution of functional redundancy may be neutral, with each community presenting the same capacity to express a similar level of redundancy under environmental forcing, with the level of functional redundancy expressed being random or influenced by external forces, which nevertheless result in comparable patterns in C substrates utilization among the different communities.

Since there is indication that metabolic plasticity appears to be intrinsic emergent community properties, likely defined by the collective genetic composition, this would suggest that microbial communities might differ in terms of their extent of functional redundancy and plasticity, with some communities being more plastic than others. In this regard, there was some indication that there may be ecosystem-specific patterns in plasticity, with lake communities presenting higher extent of both plasticity estimates (and redundancy), although the limited samples size precludes any statistical strength. These results would suggest, nevertheless, that environmental differences between the different ecosystems may select taxa that are either more functionally specialized or present a wider niche breadth, as has been previously suggested (Kirchman, 2002; Hahn, 2006; Newton et al., 2011).

On the other, our results further suggest that the metabolic plasticity as a community “trait” should be bound by the genetic composition of the dominant taxa of the community, and not by extrinsic environmental factors. This would in turn mean that the individual traits are co-selected in all the dominant taxa, but the basis for this co-selection are not known. Individual traits are defined as morphological, physiological or phenological characteristics of an organism affecting its individual performance (see Hillebrand and Matthiessen, 2009). This definition can be extended to encompass traits at the community level, and which refer to aggregate, community-level physiological or functional characteristics that influence community performance. These community-level traits influence the performance of the entire community, and in particular, the responses of these communities to environmental or biological changes, and influence the ability of these communities to cope with disturbance or stress. There is still debate as to how the individual traits scale up to determine community-level features. In the simplest scenario, these emergent community properties reflect the abundance- or biomass-weighted mean of individual traits (Diaz et al., 2007). However, there are more complex scenarios, for example, if traits are context-specific and the link between functional traits and contribution to community performance changes with changes in either the environment or in biodiversity (Fox and Harpole, 2008). Our results may offer examples of both scenarios of scaling: Metabolic plasticity appears to be an intrinsic property of the community, which is probably defined by the sum of the relative contributions of the dominant players, whereas the expression of functional redundancy appears to be more influenced by factors extrinsic to the community.

Metabolic plasticity, however, could be shaped by quite different pathways. We still do not know, for example, how metabolic plasticity is distributed among the different taxa that form the community. The null hypothesis is that the dominant taxa all have roughly the same degree of plasticity, such that the sum of the traits of these players yields a community that is also on average more plastic. The alternative hypothesis would be that bacteria within a given community vary widely in terms of their metabolic plasticity, and that the community level integrates this diversity but without reflecting the properties of any specific taxa. These two scenarios are similar in terms of the final outcome, but very different in terms of their underlying regulation and ecological significance, for whereas the first would require the co-selection of taxa that share a similar level of plasticity, the second would involve the coexistence of taxa that differ greatly in this particular trait, with the overall community property shaped by the differential expression of the trait, perhaps driven by shifts in the relative contribution of the different taxa. The fact that metabolic plasticity appears to be an intrinsic property of these bacterial communities would suggest that the former scenario is most likely.

The second major question that we explored here is whether metabolic plasticity and functional redundancy are linked across these communities, and if they are, in what manner. We found that these two community properties are strongly positively related, regardless of whether we used our “relative” or “absolute” metrics. The relationship reported in **Figure 5** therefore suggests few or no trade-offs between plasticity and redundancy, but rather co-selection of taxa that are simultaneously more metabolically plastic and which can also express a broader range of function, the latter resulting in increased functional redundancy at the community level. Trade-offs and co-selection of traits have been traditionally investigated at the strain or species level, generally focusing on functional traits (Violle et al., 2007), for example, growth capacity, tolerance to pollutants or conditions, or intrinsic characteristics such as cell size, all potentially affecting its individual performance. Trade-offs between bacterial growth and plasticity have been reported in the literature, such that bacteria that can withstand a broader range of conditions and resources tend to have low intrinsic growth rates (Hall et al., 2010).

There is a priori no reason to think that bacterial taxa that are intrinsically more metabolically plastic should also be more generalist in terms of FC. There are in fact examples in the literature of trade-offs between metabolic or growth-related traits and resource acquisition and processing capabilities in bacteria (Hall et al., 2010). For metabolic plasticity to covary positively with functional redundancy requires that when dominant taxa are intrinsically more plastic, that they would also have a broader niche breadth in terms of substrate uptake capacities. Whether these two traits tend to be co-selected, or one actually results in the other, has yet to be explored.

In summary, our experimental design together with our conceptual approach allowed us to explore how functional redundancy and metabolic plasticity vary in terms of their magnitude among communities, and also within a given community as a function of environmental forcing. We have shown that metabolic plasticity is an intrinsic emerging property of freshwater bacterialcommunities, whereas the expression of functional redundancy appears to be more strongly determined by environmental forcing. There thus appears to be a co-selection of taxa that share a certain degree of metabolic plasticity, although the underlying mechanisms are not known yet. In particular our results suggest that both redundancy and plasticity are key properties that do not necessarily shape the response of bacterial communities to the environmental forcing, but rather modulate the role that community composition and diversity play in this response. A major consequence of this tight link between redundancy and plasticity, and the fact that there is a continuum in their expression among bacterial communities, is that the apparent link between community diversity and function may also vary along a continuum, from being very tight, to being weak or even completely absent, which may in part explain the apparently conflicting results that abound in the literature. This in turn suggests that we may have to reassess not only our interpretation of current data, but also our future strategies to more effectively actually explore the role that community composition plays in the functioning of aquatic bacterial communities.

## Conflict of Interest Statement

The authors declare that the research was conducted in the absence of any commercial or financial relationships that could be construed as a potential conflict of interest.
